# Supplementary material for: Nitro-oleic acid enhances mitochondrial metabolism and ameliorates heart failure with preserved ejection fraction in mice
Source: Nat Commun. 2025 Apr 26;16:3933. doi: 10.1038/s41467-025-59192-5 (PMC12033319; doi:10.1038/s41467-025-59192-5)
Supplement: Supplementary file 1 — Supplementary Information [file 41467_2025_59192_MOESM1_ESM.pdf]

Supplementary Information

Nitro-oleic acid enhances mitochondrial metabolism and ameliorates Heart Failure with Preserved Ejection Fraction in mice

Marion Müller[1,2], Torben Schubert[1,2], Cornelius Welke[1,2], Tibor Maske[1,2], Thomas Patschkowski[3], Elfi Donhauser[1,2], Jacqueline Heinen-Weiler[4], Felix-Levin Hormann[5], Sven Heiles[5,6], Tina Johanna Schulz[1,2], Luisa Andrea Lengenfelder[1,2], Lucia Landwehrjohann[1,2], Elisa Theres Vogt[7], Bernd Stratmann[7], Jurek Hense[8], Simon Lüdtke[8], Martina Düfer[8], Elena Tolstik[5], Johann Dierks[5], Kristina Lorenz[5,9], Tamino Huxohl[10], Jan-Christian Reil[1], Vasco Sequeira[11], Francisco Jose Schopfer[12], Bruce A. Freeman[12], Volker Rudolph[1,2], Uwe Schlomann[*1,2], Anna Klinke[*#1,2]

1 Clinic for General and Interventional Cardiology/ Angiology, Herz- und Diabeteszentrum NRW, Ruhr-Universität Bochum, Bad Oeynhausen, Germany
2 Agnes Wittenborg Institute for Translational Cardiovascular Research (AWIHK), Herz- und Diabeteszentrum NRW, Ruhr-Universität Bochum, Bad Oeynhausen, Germany
3 Technology Platform Genomics, Center for Biotechnology (CeBiTec), Bielefeld University, Bielefeld, Germany
4 Medical Imaging Center (MIC), Electron Microscopy Medical Analysis – Core Facility (EMMA^{CF}), Med. Fakultät, Ruhr-Universität Bochum, Bochum, Germany
5 Leibniz-Institut für Analytische Wissenschaften-ISAS e.V., Dortmund, Germany
6 Faculty of Chemistry, University of Duisburg-Essen, 45141 Essen, Germany
7 Diabetescenter, Herz- und Diabeteszentrum NRW, Ruhr-Universität Bochum, Bad Oeynhausen, Germany
8 Institute of Pharmaceutical and Medicinal Chemistry, University of Münster, Münster, Germany
9 Institute of Pharmacology and Toxicology, University of Würzburg, Würzburg, Germany
10 Institute for Radiology, Nuclear Medicine and Molecular Imaging, Herz- und Diabeteszentrum NRW, Ruhr-Universität Bochum, Bad Oeynhausen, Germany
11 Department of Translational Science Universitätsklinikum, DZHI, Würzburg, Germany
12 Department of Pharmacology and Chemical Biology, University of Pittsburgh, Pittsburgh, PA, USA.

* contributed equally

[#] Address for correspondence
Anna Klinke, PhD
Clinic for General and Interventional Cardiology/ Angiology
Herz- und Diabeteszentrum NRW
University Hospital, Ruhr Universität Bochum
Georgstrasse 11, 32545 Bad Oeynhausen, Germany
E-Mail: aklinke@hdz-nrw.de
Tel.: +49-5731-973668

Supplementary Figures and Legends

Figure S1

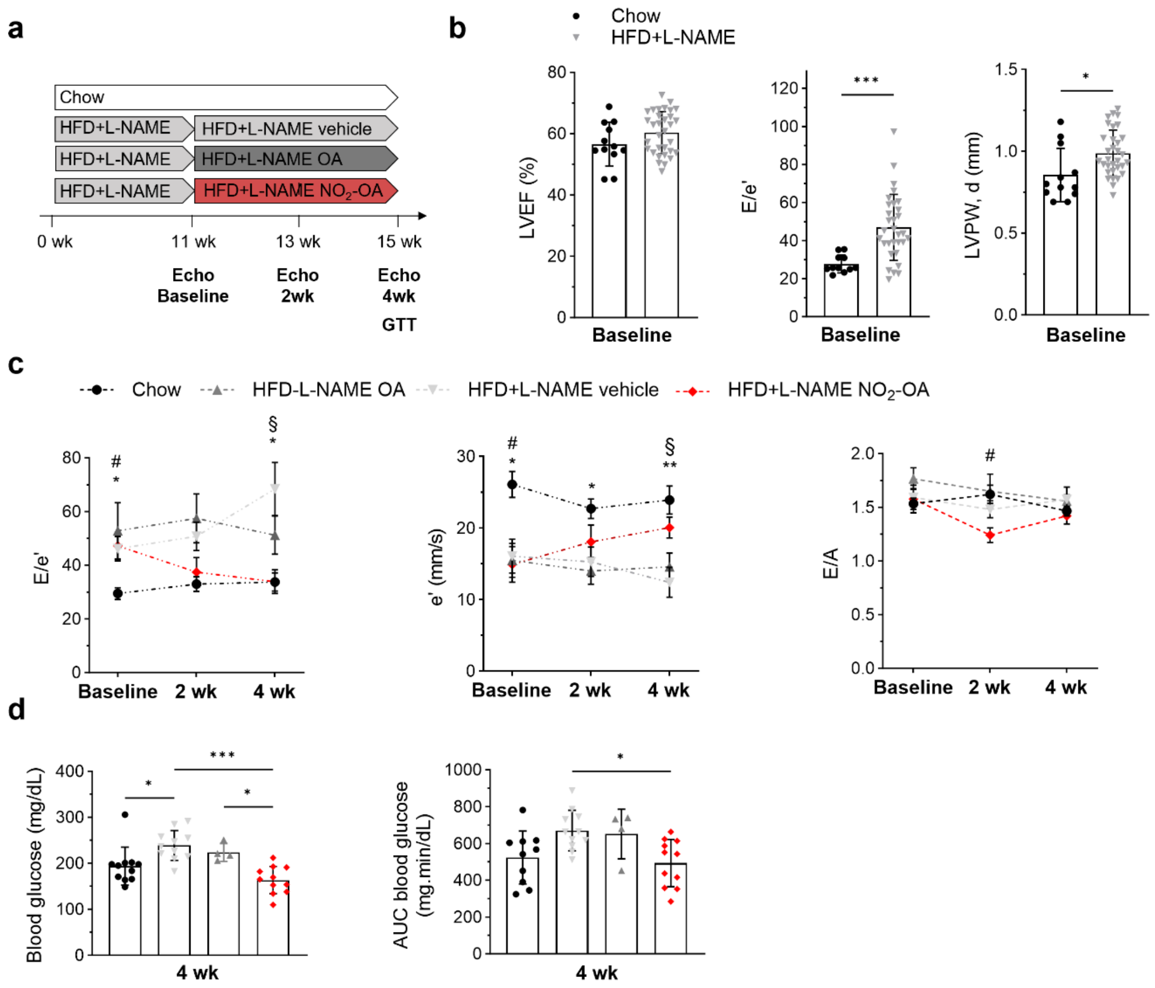

Figure S1: Nitro-oleic acid but not oleic acid has key impact on cardiac function and glucose tolerance.
a Mice received high-fat diet and the endothelial nitric oxide synthase inhibitor L-NAME (HFD+L-NAME) or chow diet (Chow) for 15 weeks (wk) and were treated with vehicle, nitro-oleic acid (NO_2-OA) or oleic acid (OA) for the last 4 wk. Echocardiography (Echo) was performed after 11 wk of chow or HFD+L-NAME (Baseline) and after 15 wk of chow or HFD+L-NAME including 4 wk of vehicle-, OA- or NO_2-OA-treatment. Glucose tolerance test (GTT) was conducted at the final time point (4 wk). **b** Left ventricular ejection fraction (LVEF), ratio of early diastolic mitral inflow velocity to early diastolic mitral annulus velocity (E/e´) and left ventricular posterior wall thickness (LVPW, d) after 11 wk of chow or HFD+L-NAME (Baseline) (N=12/32). **c** Trend of E/e´ ratio, e' and E/A ratio during treatment of HFD+L-NAME mice with vehicle, OA or NO_2-OA compared to chow mice (N=12/10/11/10). **d** Blood glucose levels during an intraperitoneal glucose tolerance test shown as blood glucose concentrations 90 min after glucose injection (N=11/11/4/11) and area under the curve (AUC) over the total time of 90 min (N=10/11/4/11). Data are mean ± standard deviation for **b** and **d.** **c** is shown as mean ± standard error of the mean. Statistical significance was calculated by unpaired, two-sided Student's t-test for **b** and mixed effect analysis followed by Bonferroni´s multiple comparison test in **c**. * indicates statistical significance between chow and untreated HFD+L-NAME. # indicates statistical significance between chow and NO_2-OA treated HFD+L-NAME. § indicates statistical significance between NO_2-OA treated and untreated HFD+L-NAME. Statistical significance in **d** was calculated by Kruskal-Wallis test followed by Dunn´s multiple comparisons test for raw blood glucose values and One-way ANOVA followed by Bonferroni´s post-hoc test for AUC. Only statistically significant differences are indicated. p: *<0.05, **<0.01, ***<0.001. N represent individual animals. Variation in N is due to failed blood collection. Outliers were identified using ROUT method and excluded from analysis.

Figure S2

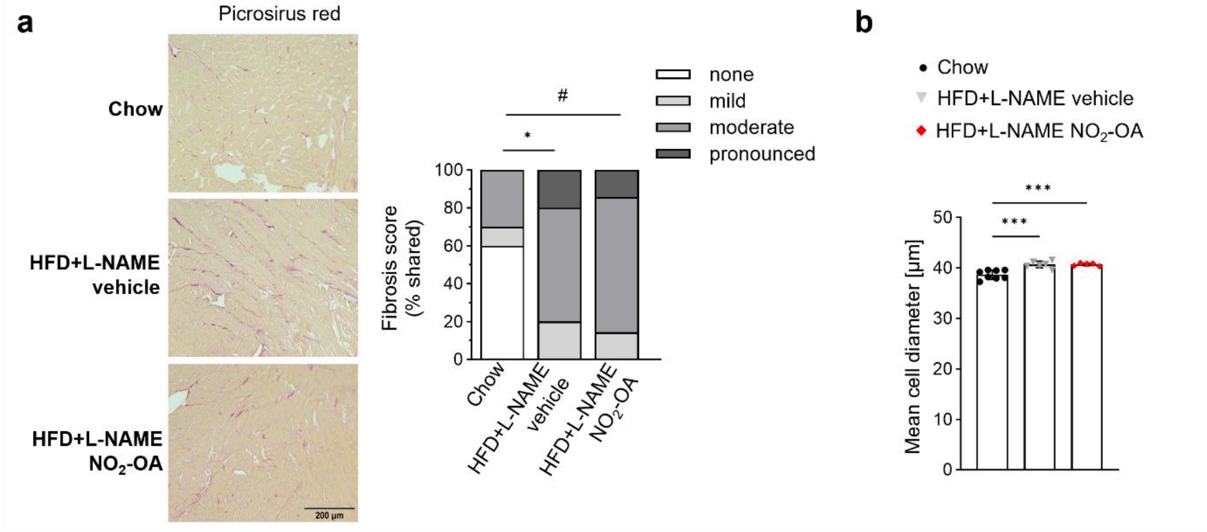

Figure S2: Nitro-oleic acid has no effect on cardiac structural remodeling in HFpEF mice.
a Representative images (scale bar 200 µm) of LV sections stained with picrosirius red and analysis of fibrosis grades (N=10/10/7). Images were scored based on the amount of collagen deposition. **b** Mean cell diameter of isolated cardiomyocytes assessed by pulse area analysis-based volumetry (N=8/6/5). Data are mean ± standard deviation for **b**. Statistical significance was calculated by Chi square test for **a**. * presents analysis of chow vs. HFD+L-NAME vehicle and # analysis of HFD+L-NAME vehicle vs. NO_2-OA group. One-way ANOVA with Bonferroni´s post-hoc test was used for **b**. Only statistically significant differences are indicated. p: *<0.05, ***<0.001. N represent individual animals.

Figure S3

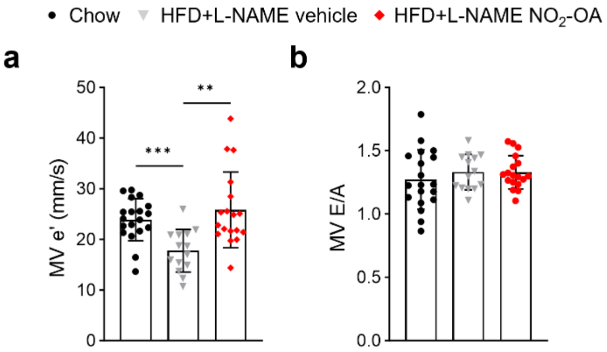

Figure S3: Diastolic function is improved in nitro-oleic acid-treated HFpEF mice.
a Early diastolic mitral annulus velocity (e') was assessed by echocardiography using tissue doppler. **b** The trans mitral profile (E/A ratio) was assessed by pulse wave doppler. (N=19/14/18) Data are mean ± standard deviation. Statistical significance was calculated by One-way ANOVA with Bonferroni´s post-hoc test. Only statistically significant differences are indicated. p: **<0.01, ***<0.001. N represent individual animals.

Figure S4

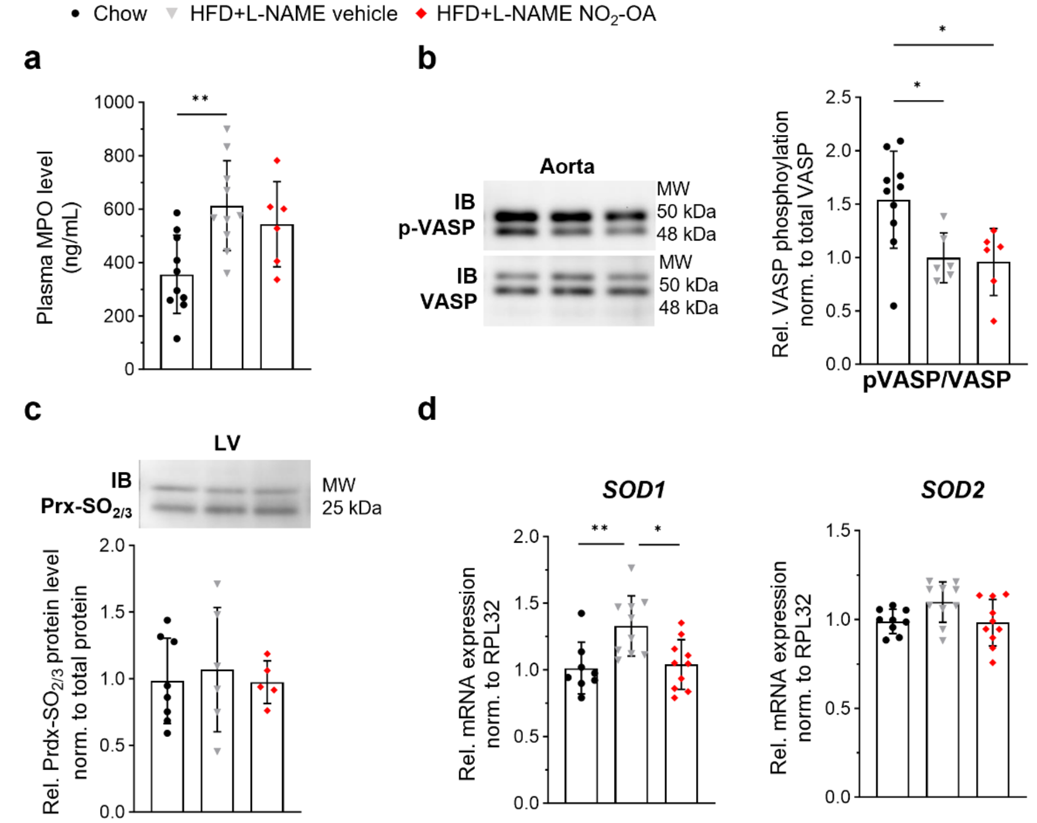

Figure S4: Nitro-oleic acid has no effect on systemic inflammation, vascular nitric oxide bioavailability and oxidative stress.

a Plasma level of myeloperoxidase (MPO) (N=10/10/6). **b** Level of phosphorylation (Ser239, pVASP) of the vasodilator-stimulated phosphoprotein (VASP) was quantified via immunoblot (N=10/6/6) in aorta. **c** Representative immunoblot and quantification of hyperoxidized peroxiredoxin (Prdx-$SO_{2/3}$) in left-ventricular mouse tissue (N=8/6/5). **d** mRNA level of superoxide dismutase 1 (*SOD1*) and superoxide dismutase 2 (*SOD2*) (N=9/10/10). Data are mean ± standard deviation Statistical significance was calculated by One-way ANOVA followed by Bonferroni´s post-hoc test. Only statistically significant differences are indicated. p: *<0.05, **<0.01. N represent individual animals.

Figure S5

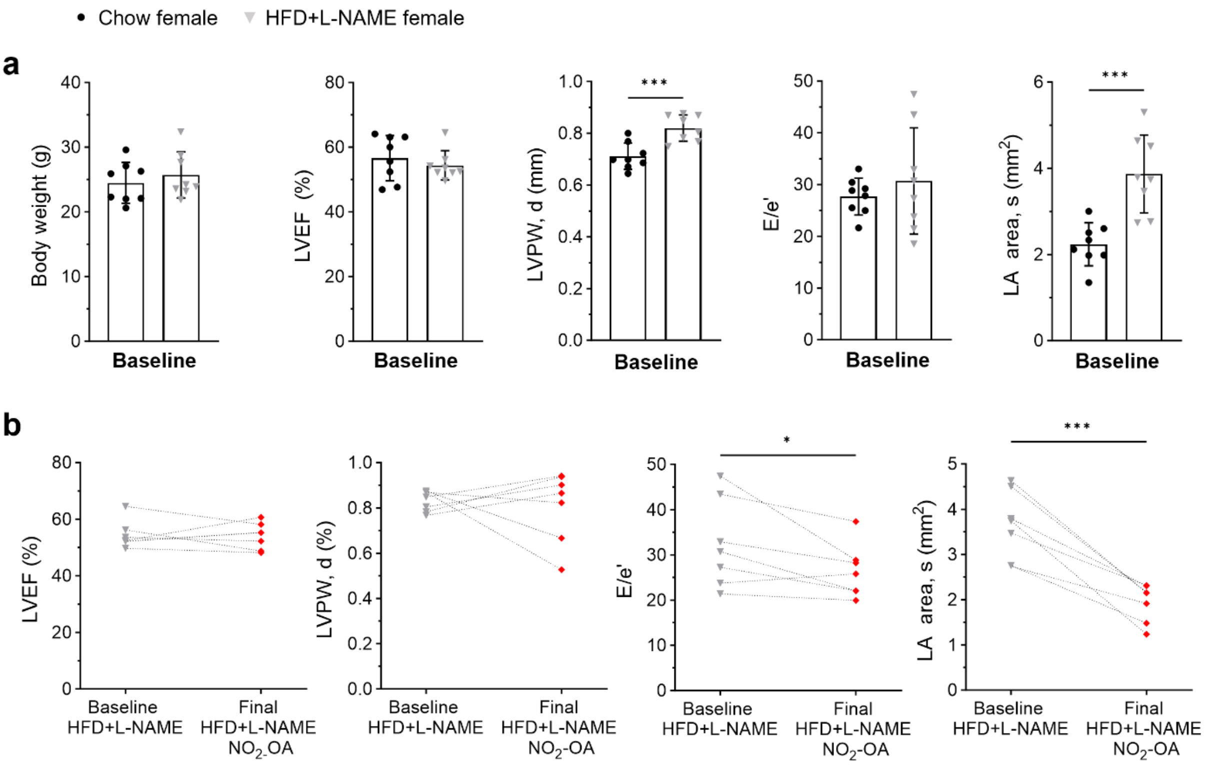

Figure S5: Effects of nitro-oleic acid on heart function could be confirmed in female mice with metabolic cardiomyopathy.
a 8 week (wk) old female mice received high-fat diet and the endothelial nitric oxide synthase inhibitor L-NAME (HFD+L-NAME) or chow diet (Chow). After 15 wk heart function was evaluated by echocardiography (Baseline) (N=8/8). **b** All HFD+L-NAME mice were treated with NO_2-OA for 4 wk and echocardiography was performed at the final timepoint (Final). One mouse refused oral administration of NO_2-OA (N=7/7). Data are mean ± standard deviation for **a**. Individual animals are shown in **b.** Statistical significance was calculated by unpaired Student´s t-test for **a** and paired Student's t-test for **b**. Only statistically significant differences are indicated. p: *<0.05, ***<0.001. LVEF, left ventricular ejection fraction; LVPW, d, LV posterior wall thickness; E/e´, ratio of early diastolic mitral inflow velocity to early diastolic mitral annulus velocity; LA area, s, left atrial area at systole.

Figure S6

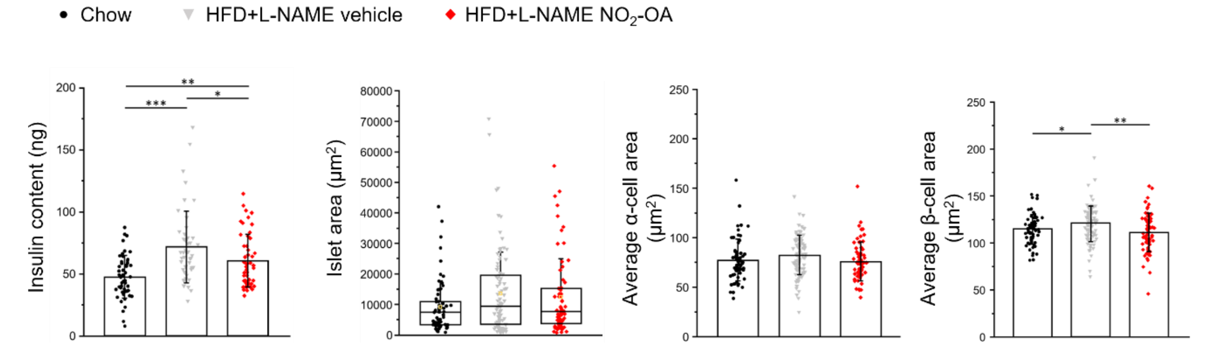

Figure S6: Nitro-oleic acid partly counteracts the changes of the endocrine pancreas induced by HFD+L-NAME.
Insulin content was determined per islet (10/10/10 animals per cohort) and analysis of whole islet and the average α- or β-cell size was performed in pancreatic tissue slices. Parameters are calculated as area per islet and cell, respectively (5/5/4 animals per cohort). One-way ANOVA followed by Neumann-Keuls post-hoc test was used for insulin content and the average cell area of α and β-cells. Kruskal-Wallis test followed by Dunn´s multiple comparisons was used for the islet area. Only statistically significant differences are indicated. p: *<0.05, **<0.01, ***<0.001. N represent islets.

Figure S7

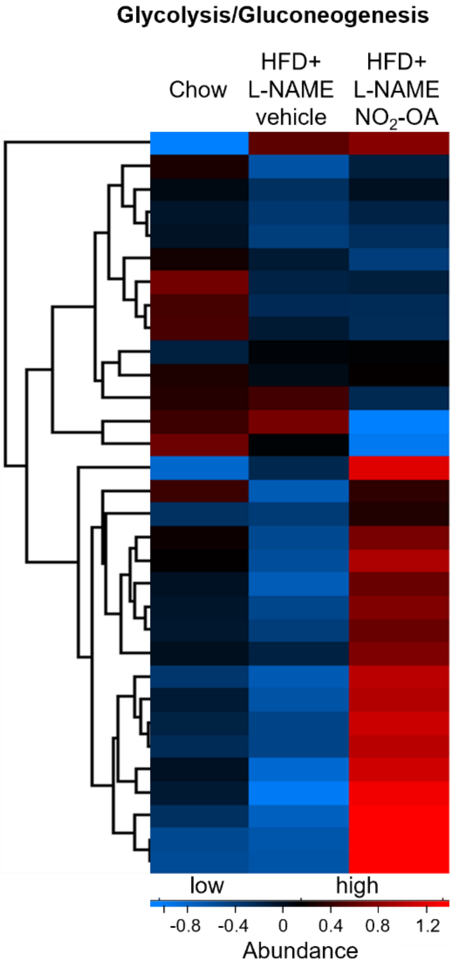

Figure S7: Nitro-oleic acid induces a beneficial metabolic protein profile in LV of mice with HFpEF. Heatmap presenting the protein abundance of all proteins associated with the KEGG pathway glycolysis/gluconeogenesis for left ventricular tissue of mice after chow diet, 15 weeks (wk) of HFD and L-NAME with 4 wk of vehicle (HFD+L-NAME vehicle) or NO_2-OA treatment (HFD+L-NAME NO_2-OA) obtained by liquid chromatography – mass spectrometry (N=9/8/6). The protein names and z-scores are presented in the source data file.

Figure S8

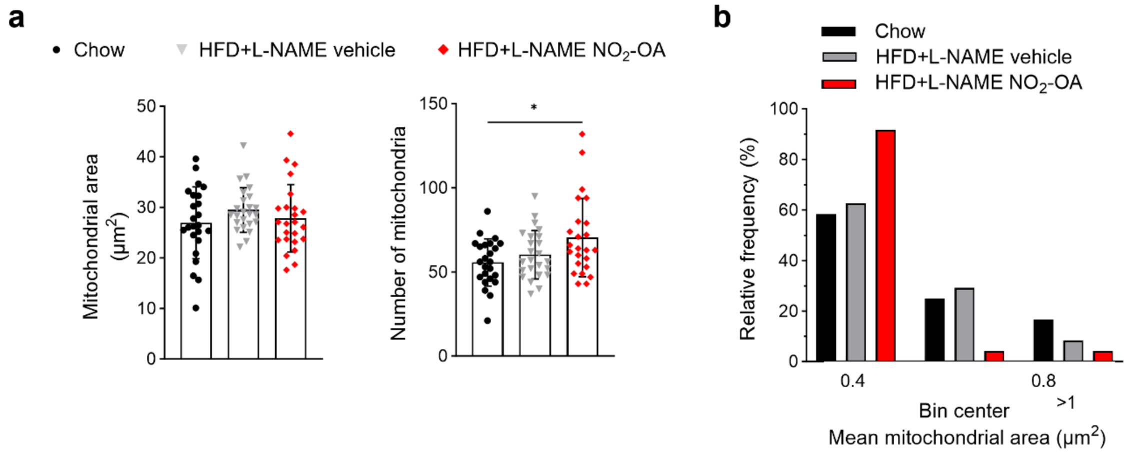

Figure S8: Nitro-oleic acid treatment leads to smaller but markedly increased number of mitochondria in HFpEF mice.

a Quantitative analysis of mitochondrial area in µm^2 (left) and number of mitochondria (right). **b** Histogram presenting the distribution of the mean mitochondrial area in µm^2 per quantified image. Data are mean ± standard deviation for **a**. Statistical significance was calculated with One-way ANOVA followed by Bonferroni´s post-hoc test. Only statistically significant differences are indicated. p: *<0.05. N represent individual transmission electron microscopy images (24 images per group from N=4/5/4 animals).

Figure S9

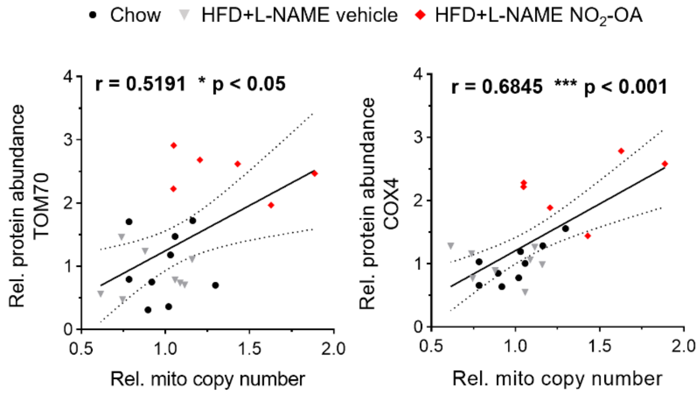

Figure S9: Mitochondrial protein level correlates with mitochondrial copy number.

Correlation of mitochondrial protein level, reflected by level of the translocase of outer mitochondrial membrane 70 (TOM70) and cytochrome c oxidase 4 (COX4), with relative mitochondrial copy number of left ventricular mouse tissue (N=9/8/6). Statistical significance was calculated by Pearson correlation. p: *<0.05, ***<0.001. N represent individual animals.

Figure S10

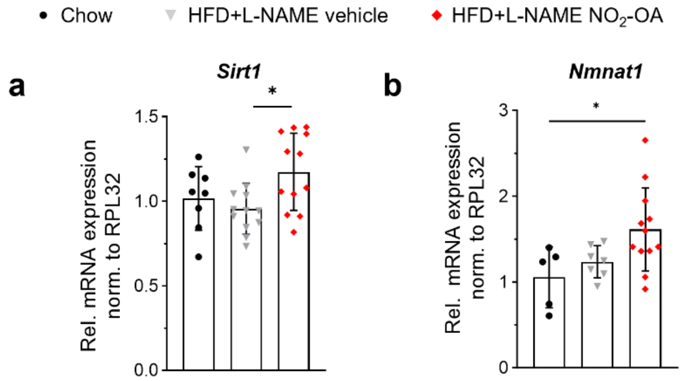

Figure S10: Downstream targets of AMPK signaling are increased by nitro-oleic acid in HFpEF mice. mRNA expression analyses of the NAD-dependent protein deacetylase sirtuin-1 (*Sirt1*) (N=8/11/12) (**a**) and the nicotinamide nucleotide adenyl transferase 1 (*Nmnat1*) (N=5/7/12) (**b**) were performed in left ventricular mouse tissue. Data are mean ± standard deviation. Statistical significance was calculated with One-way ANOVA followed by Bonferroni´s post-hoc test. Only statistically significant differences are indicated. p: *<0.05. N represent individual animals.

Figure S11

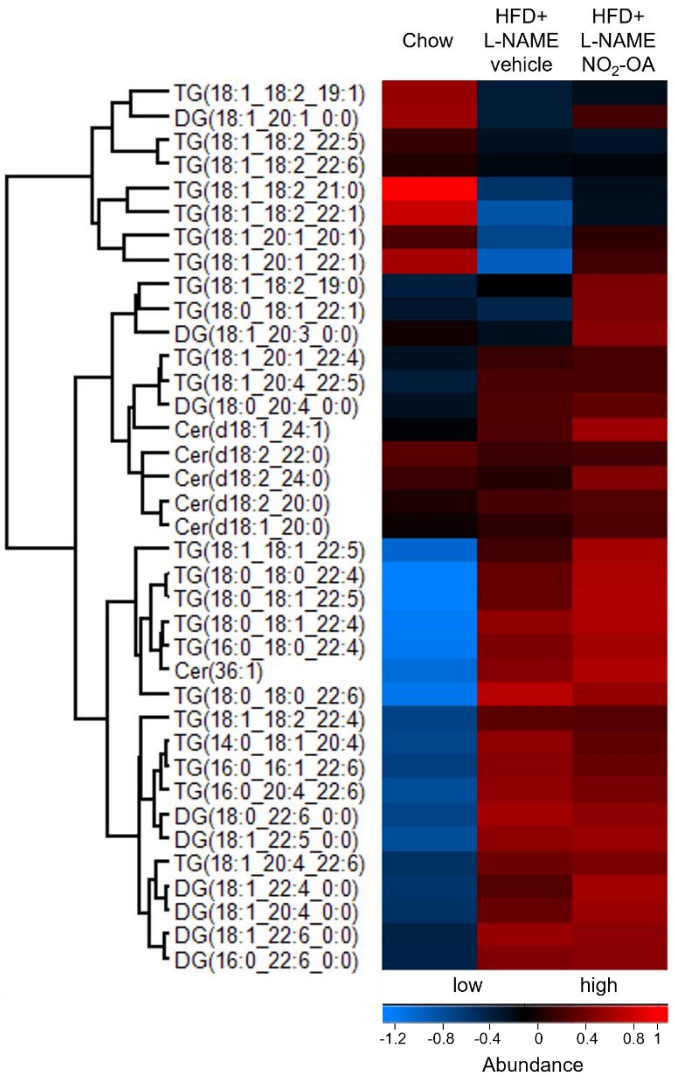

Figure S11: Lipid analysis of myocardial tissue reveals augmented levels of diacylglycerols, triacylglycerols and ceramides in the HFD+L-NAME groups.
Heatmap presenting the diacylglycerols (DG), triacylglycerols (TG) and ceramides (Cer), which contain fatty acids that were provided by feeding the animals with HFD, detected in septal tissue of mice after chow diet, 15 weeks (wk) of HFD and L-NAME with 4 wk of vehicle (HFD+L-NAME vehicle) or NO_2-OA treatment (HFD+L-NAME NO_2-OA) obtained by lipid chromatography mass spectrometry (N=5/5/5). Lipid species and z-scores are listed in the source data file and data for all analysed lipid species are listed in Table S2.

Figure S12

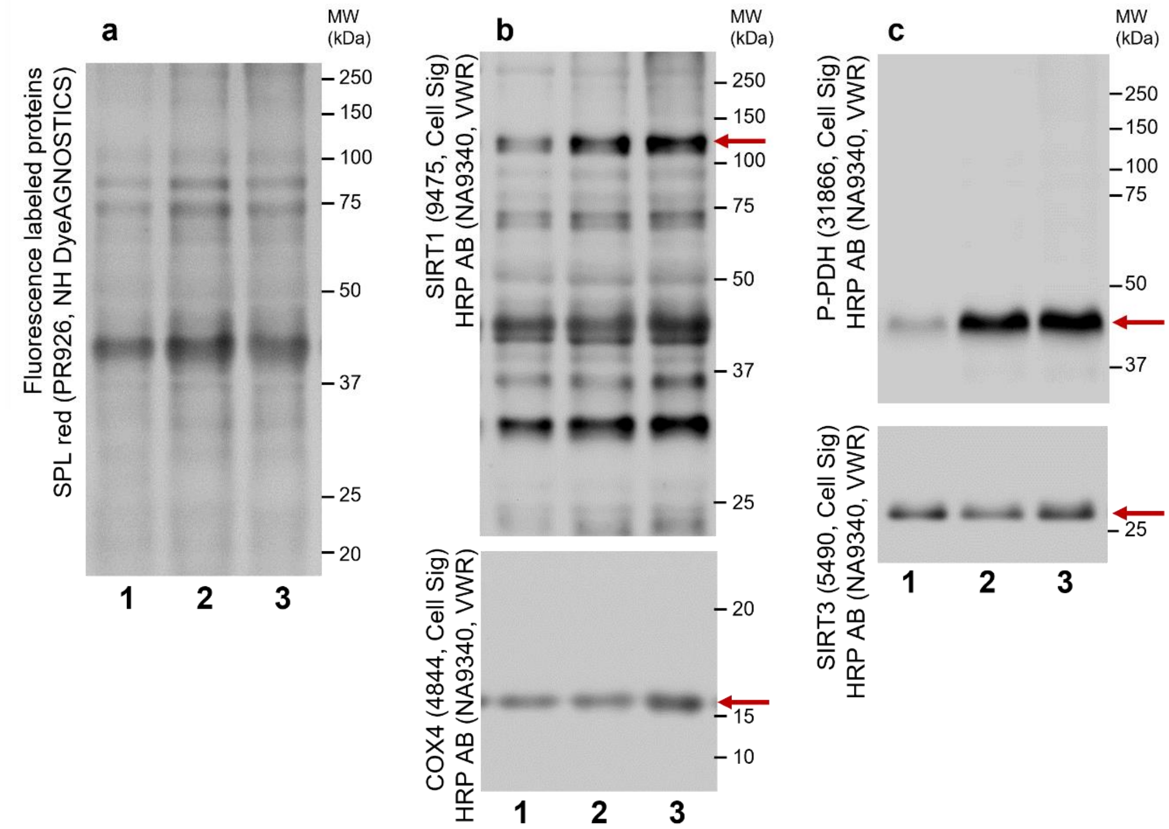

Figure S12: Detection of protein level in left ventricular tissue of control and HFpEF mice. Mice received high-fat diet (HFD) and the endothelial nitric oxide synthase inhibitor L-NAME or chow diet (chow) (N=9) for 15 weeks (wk) and were treated with nitro-oleic acid (NO_2-OA) (N=6) or vehicle (N=8) for 4 wk (wk 12 to 15). Proteins were isolated and used for proteome analysis and immunoblot. Representative immunoblots are shown for the three experimental groups (1: chow; 2: HFD+L-NAME vehicle; 3: HFD+L-NAME NO_2-OA). **a** Proteins were labeled with SPL red to assess total protein amount by fluorescence. **b** The membrane was cut and incubated with sirtuin 1 (SIRT1) (above) or cytochrome c oxidase subunit 4 (COX4) (down) primary antibody. After incubation with HRP-conjugated secondary antibody the protein level was assessed by chemiluminescence. **c** Reblot of membrane and second incubation with antibody detecting the phosphorylation at serine 293 of pyruvate dehydrogenase (P-PDH) (above) or sirtuin 3 (SIRT3) (down). After incubation with HRP-conjugated secondary antibody the protein level was assessed by chemiluminescence.

Figure S13

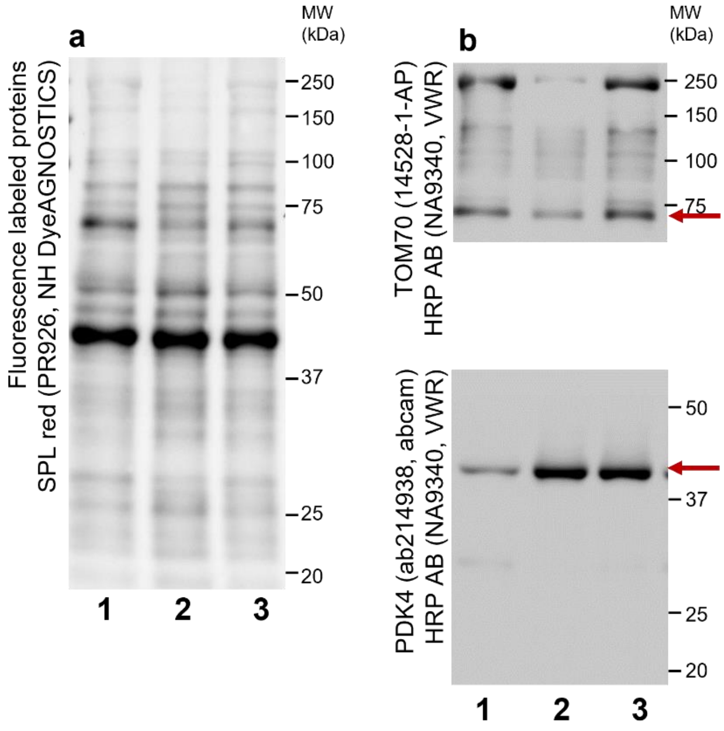

Figure S13: Detection of protein level in left ventricular tissue of control and HFpEF mice.
Mice received high-fat diet (HFD) and the endothelial nitric oxide synthase inhibitor L-NAME or chow diet (chow) (N=9) for 15 weeks (wk) and were treated with nitro-oleic acid (NO_2-OA) (N=6) or vehicle (N=8) for 4 wk (wk 12 to 15). Proteins were isolated and used for proteome analysis and immunoblot. Representative immunoblots are shown for the three experimental groups (1: chow; 2: HFD+L-NAME vehicle; 3: HFD+L-NAME NO_2-OA). **a** Proteins were labeled with SPL red to assess total protein amount by fluorescence. **b** The membrane was incubated with antibody against translocase of outer mitochondrial membrane 70 (TOM70) (above) or pyruvate dehydrogenase kinase 4 (PDK4) (down). After incubation with HRP-conjugated secondary antibody the protein level was assessed by chemiluminescence.

Figure S14

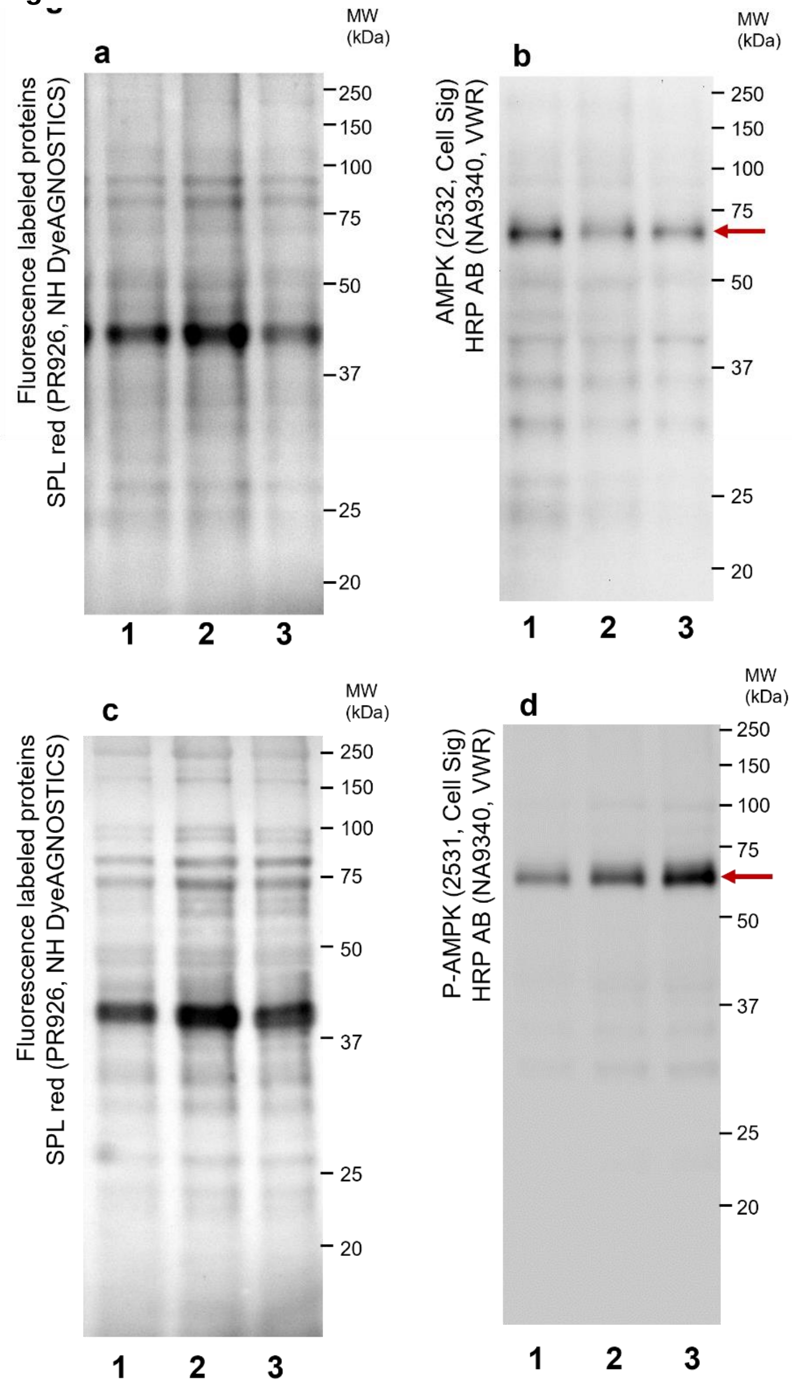

Figure S14: Detection of protein level in left ventricular tissue of control and HFpEF mice. Mice received high-fat diet (HFD) and the endothelial nitric oxide synthase inhibitor L-NAME or chow diet (chow) (N=9) for 15 weeks (wk) and were treated with nitro-oleic acid (NO_2-OA) (N=6) or vehicle (N=8) for 4 wk (wk 12 to 15). Proteins were isolated and used for proteome analysis and immunoblot. Representative immunoblots are shown for the three experimental groups (1: chow; 2: HFD+L-NAME vehicle; 3: HFD+L-NAME NO_2-OA). **a**, **c** Proteins were labeled with SPL red to assess total protein amount by fluorescence. **b** The membrane was incubated with AMP-activated protein kinase (AMPK) primary antibody. After incubation with HRP-conjugated secondary antibody the protein level was assessed by chemiluminescence. **d** The membrane was incubated with antibody detecting the phosphorylation at threonine 172 of AMP-activated protein kinase (AMPK). After incubation with HRP-conjugated secondary antibody the protein level was assessed by chemiluminescence.

Figure S15

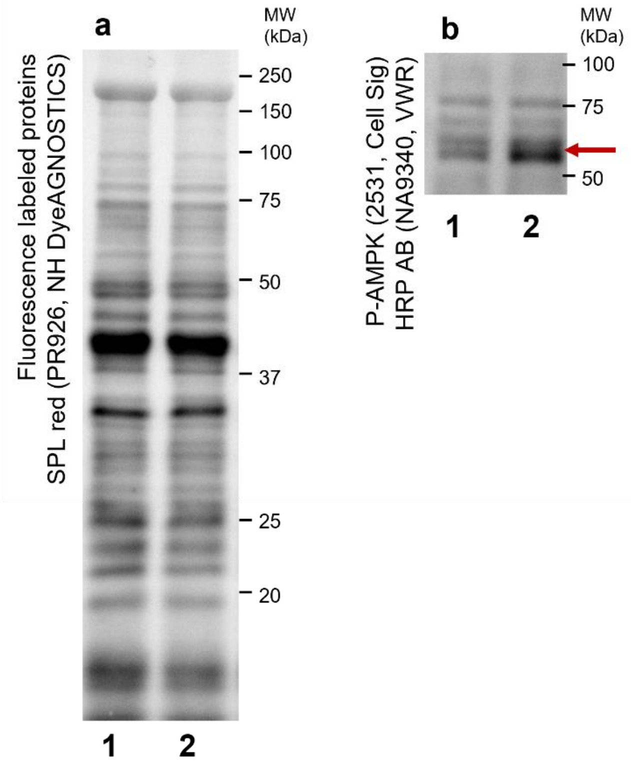

Figure S15: Detection of protein level in metabolically-stressed cardiomyocytes.
Isolated adult murine cardiomyocytes were cultivated for 48 hrs under metabolic stress conditions induced by high glucose levels (high Glu), endothelin-1 (ET-1) and hydrocortisone (HC) with treatment of methanol (Vehicle) or nitro-oleic acid (NO_2-OA). Representative immunoblots are shown for the two experimental groups (1: high Glu, ET-1, HC vehicle; 2: high Glu, ET-1, HC NO_2-OA). **a** Proteins were labeled with SPL red to assess total protein amount by fluorescence. **b** The membrane was cut and incubated with antibody detecting the phosphorylation at threonine 172 of AMP-activated protein kinase (AMPK). After incubation with HRP-conjugated secondary antibody the protein level was assessed by chemiluminescence.

Figure S16

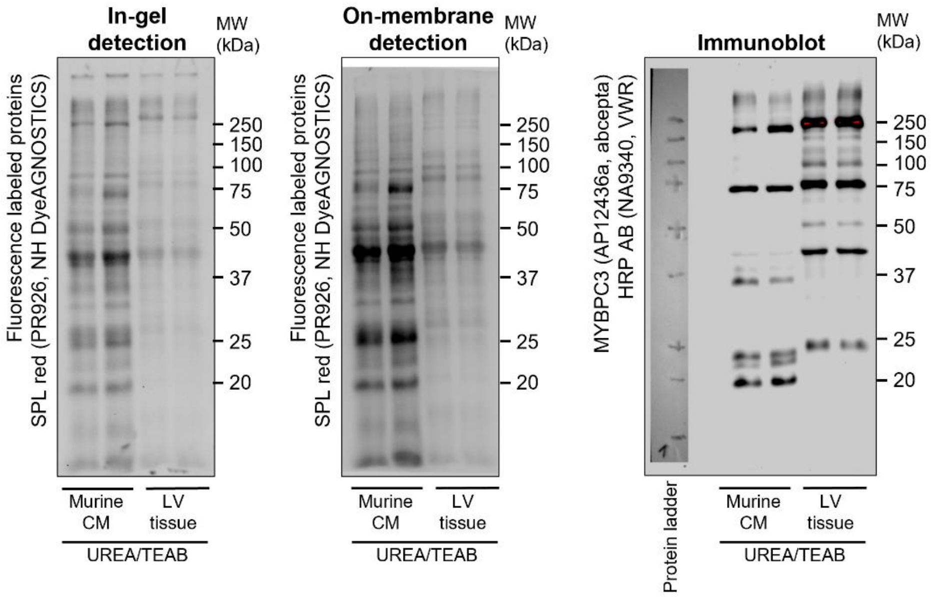

Figure S16: Detection of high-molecular weight proteins in left ventricular mouse tissue (LV tissue) and murine cardiomyocytes (Murine CM) isolated with UREA/TEAB buffer.

Total proteins were detected by fluorescence signal after staining with SPL red before (In-gel-detection) or after blotting (On-membrane detection) on PVDF membrane. The membrane was stained with antibodies against the myosin binding protein C (MYBPC3).

Contents of Report

Created by https://lipidomicstandards.org, version v2.4.0

Separation Workflow 1
Overall study design . . . 1
Lipid extraction . . . 1
Analytical platform . . . 1
Quality control . . . 1
Method qualification and validation . . . 2
Reporting . . . 2

Sample Descriptions 2
Heart septa, chow diet / Mouse / Tissues (e.g., liver, heart, brain) . . . 2
Heart septa, HFD+L-NAME, vehicle / Mouse / Tissues (e.g., liver, heart, brain) . . . 2
Heart septa, HFD+L-NAME, NO2-OA / Mouse / Tissues (e.g., liver, heart, brain) . . . 3

Separation Workflow

Overall study design

Title of the study	Enhanced cardiac mitochondrial biogenesis by nitro-oleic acid remedies diastolic dysfunction in a mouse model of heart failure with preserved ejection fraction		
Document creation date	03/24/2025	Corresponding Email	sven.heiles@isas.de
Principal investigator	Sven Heiles	Is the workflow targeted or untargeted?	Untargeted
Institution	Leibniz-Institut für Analytische Wissenschaften - ISAS - e.V.	Clinical	No

Lipid extraction

Extraction method	2-phase system	Were internal standards added prior extraction?	Yes
pH adjustment	None	Special conditions	extraction was performed at temperatures of/or below 4 °C
2-phase system	MTBE	Derivatization	-

Analytical platform

Ionizaton additives	Ammonium formate, Formic acid	Resolution at m/z 200 at MS1	180000
Number of separation dimensions	One dimension	Mass accuracy in ppm at MS1	3
Separation type 1	LC	Recording mode of raw data at MS1	Profile mode
Separation mode 1 (liquid)	RP	Mass window for precursor ion isolation (in Da total isolation window)	2
Detector	Mass spectrometer	Mass resolution for detected ion at MS2	High resolution
MS type	Orbitrap	Resolution at m/z 200 at MS2	15000
MS vendor	Thermo	Mass accuracy in ppm at MS2	5
Ion source	HESI	Recording mode of raw data at MS2	Profile mode
MS Level	MS1, MS2	Was/Were additional dimension/techniques used	No
Mass resolution for detected ion at MS1	High resolution		

Quality control

Blanks	Yes	Quality control	Yes
Type of Blanks	Extraction blank, Solvent blank	Type of QC sample	Sample pool

Method qualification and validation

Method validation	No

Reporting

Are reported raw data uploaded into repository?	No	Raw data upload	Available on request
Are metadata available?	Available on request	Additional comments	-

Sample Descriptions

Heart septa, chow diet / Mouse / Tissues (e.g., liver, heart, brain)

Perfusion	No	Additives	None
Storage and collection conditions	Available	Were samples stored under inert gas?	No
Provided preanalytical information	Storage time (month)	Additional preservation methods	No
Temperature handling original sample	4-8 °C	Biobank samples	No
Instant sample preparation	No	Sample homogenization	Yes
Storage temperature	-80 °C	Sample homogenization solvent	0.1 % ammoniumacetate in water
Storage time (month)	3		

Heart septa, HFD+L-NAME, vehicle / Mouse / Tissues (e.g., liver, heart, brain)

Perfusion	No	Additives	None
Storage and collection conditions	Available	Were samples stored under inert gas?	No
Provided preanalytical information	Storage time (month)	Additional preservation methods	No
Temperature handling original sample	4-8 °C	Biobank samples	No
Instant sample preparation	No	Sample homogenization	Yes
Storage temperature	-80 °C	Sample homogenization solvent	0.1 % ammoniumacetate in water
Storage time (month)	3		

Heart septa, HFD+L-NAME, NO2-OA / Mouse / Tissues (e.g., liver, heart, brain)

Perfusion	No	Additives	None
Storage and collection conditions	Available	Were samples stored under inert gas?	No
Provided preanalytical information	Storage time (month)	Additional preservation methods	No
Temperature handling original sample	4-8 °C	Biobank samples	No
Instant sample preparation	No	Sample homogenization	Yes
Storage temperature	-80 °C	Sample homogenization solvent	0.1 % ammoniumacetate in water
Storage time (month)	3		

Lipid Class Descriptions

1) DG[M+NH4]+ / Lipid identification

Lipid class	DG	Did you presume assumptions for identification?	No
MS Level for identification	MS1, MS2	Check on:	-
Identification level	Molecular species level	Limit of detection	No
Polarity mode	Positive	RT verified by standard	Yes
Type of positive (precursor)ion	[M+NH4]+	Separation of isobaric/isomeric interferece confirmed	No
Fragments for identification<br>**Fragment name**<br>-FA2(-H)-(H2O+NH3)		Model for separation prediction	No
Isotope correction at MS1	No	Additional dimension/techniques	-
Isotope correction at MS2	No	Lipid Identification Software	Lipostar2
MS1 verified by standard	Yes	Data manipulation	Smoothing, Background subtraction
MS2 verified by standard	No	Nomenclature for intact lipid molecule	Yes
Background check at MS1	No	Nomenclature for fragment ions	Yes
Background check at MS2	No	Further identification remarks	-

1) DG[M+NH4]+ / Lipid quantification

Quantitative	No	Batch correction	No
Normalization to reference	Yes	Further quantification remarks	-

2) TG[M+NH4]+ / Lipid identification

Lipid class	TG	Did you presume assumptions for identification?	No
MS Level for identification	MS1, MS2	Check on:	-
Identification level	Molecular species level	Limit of detection	No
Polarity mode	Positive	RT verified by standard	Yes
Type of positive (precursor)ion	[M+NH4]+	Separation of isobaric/isomeric interferece confirmed	No
Fragments for identification		Model for separation prediction	No

Fragment name
-FA3(+HO)-(NH3)
-FA1(+HO)-(NH3)

Isotope correction at MS1	No	Additional dimension/techniques	-
Isotope correction at MS2	No	Lipid Identification Software	Lipostar2
MS1 verified by standard	Yes	Data manipulation	Smoothing, Background subtraction
MS2 verified by standard	No	Nomenclature for intact lipid molecule	Yes
Background check at MS1	No	Nomenclature for fragment ions	Yes
Background check at MS2	No	Further identification remarks	-

2) TG[M+NH4]+ / Lipid quantification

Quantitative	No	Batch correction	No
Normalization to reference	Yes	Further quantification remarks	-

3) Cer[M+H]+ / Lipid identification

Lipid class	Cer	Did you presume assumptions for identification?	No
MS Level for identification	MS1, MS2	Check on:	-
Identification level	Molecular species level	Limit of detection	No
Polarity mode	Positive	RT verified by standard	Yes
Type of positive (precursor)ion	[M+H]+	Separation of isobaric/isomeric interferece confirmed	No
Fragments for identification		Model for separation prediction	No

Fragment name
LCB(-H3O2)

Isotope correction at MS1	No	Additional dimension/techniques	-
Isotope correction at MS2	No	Lipid Identification Software	Lipostar2
MS1 verified by standard	Yes	Data manipulation	Smoothing, Background subtraction
MS2 verified by standard	No	Nomenclature for intact lipid molecule	Yes
Background check at MS1	No	Nomenclature for fragment ions	Yes
Background check at MS2	No	Further identification remarks	-

3) Cer[M+H]+ / Lipid quantification

Quantitative	No	Batch correction	No
Normalization to reference	Yes	Further quantification remarks	-
