# Supplementary material for: Nitro-oleic acid enhances mitochondrial metabolism and ameliorates heart failure with preserved ejection fraction in mice
Source: Nat Commun. 2025 Apr 26;16:3933. doi: 10.1038/s41467-025-59192-5 (PMC12033319; doi:10.1038/s41467-025-59192-5)
Supplement: Supplementary file 3 — Description of Additional Supplementary Files [file 41467_2025_59192_MOESM3_ESM.pdf]

Description of Additional Supplementary Files

Supplementary Data 1: Liquid-chromatography mass spectrometry data for proteome analysis derived from left ventricular tissue of 9 control mice (Chow), 8 mice, that received high-fat diet and the eNOS inhibitor L-NAME for 15 weeks (wk) and were treated with vehicle for the last 4 wk (HFD+L-NAME vehicle), and 6 HFD+L-NAME mice, that were treated with NO2-OA for the last 4 wk (HFD+L-NAME NO2-OA). Data were processed with the Proteome Discoverer 2.5 software (Thermo Fisher, Dreieich, Germany). No statistics, which test differences between experimental groups, were applied.

Supplementary Data 2: Liquid-chromatography mass spectrometry data for lipid analysis displaying the different lipid species for every sample (Chow: N=5; HFD+L-NAME vehicle: N=5; HFD+L-NAME NO2-OA: N=5). No statistics, which test differences between experimental groups, were applied.
