# Supplementary material for: Nitro-oleic acid enhances mitochondrial metabolism and ameliorates heart failure with preserved ejection fraction in mice
Source: Nat Commun. 2025 Apr 26;16:3933. doi: 10.1038/s41467-025-59192-5 (PMC12033319; doi:10.1038/s41467-025-59192-5)
Supplement: Supplementary file 6 — Reporting Summary [file 41467_2025_59192_MOESM6_ESM.pdf]

nature portfolio

Corresponding author(s): Anna Klinke

Last updated by author(s): Mar 19, 2025

Reporting Summary

Nature Portfolio wishes to improve the reproducibility of the work that we publish. This form provides structure for consistency and transparency in reporting. For further information on Nature Portfolio policies, see our Editorial Policies and the Editorial Policy Checklist.

Statistics

For all statistical analyses, confirm that the following items are present in the figure legend, table legend, main text, or Methods section.

n/a	Confirmed	
☐	☒	The exact sample size (*n*) for each experimental group/condition, given as a discrete number and unit of measurement
☐	☒	A statement on whether measurements were taken from distinct samples or whether the same sample was measured repeatedly
☐	☒	The statistical test(s) used AND whether they are one- or two-sided<br>*Only common tests should be described solely by name; describe more complex techniques in the Methods section.*
☒	☐	A description of all covariates tested
☐	☒	A description of any assumptions or corrections, such as tests of normality and adjustment for multiple comparisons
☐	☒	A full description of the statistical parameters including central tendency (e.g. means) or other basic estimates (e.g. regression coefficient) AND variation (e.g. standard deviation) or associated estimates of uncertainty (e.g. confidence intervals)
☐	☒	For null hypothesis testing, the test statistic (e.g. *F*, *t*, *r*) with confidence intervals, effect sizes, degrees of freedom and *P* value noted<br>*Give P values as exact values whenever suitable.*
☒	☐	For Bayesian analysis, information on the choice of priors and Markov chain Monte Carlo settings
☒	☐	For hierarchical and complex designs, identification of the appropriate level for tests and full reporting of outcomes
☐	☒	Estimates of effect sizes (e.g. Cohen's *d*, Pearson's *r*), indicating how they were calculated

Our web collection on statistics for biologists contains articles on many of the points above.

Software and code

Policy information about availability of computer code

Data collection	Vevo 3100 Imaging System (FUJIFILM Visualsonics, Inc., Toronto, ON, Canada) using a MX550D transducer (25 55 MHz, FUJIFILM Visualsonics, Inc., Toronto, ON, Canada); ADInstruments PowerLab C system (ADInstruments, Dunedin, New Zealand); Panlab treadmill (Model: LE8708TS Airtight, Panlab, Harvard Apparatus, Holliston, MA, USA); Nano lipid chromatograph (Ultimate 3000, Thermo Fisher Scientific, Germany) coupled to a modified ESI-Orbitrap MS/MS (QExactive Plus, Thermo Scientific™, Walthem, MA, USA) equipped with a Spectroglyph source (Spectroglyph, LLC, WA, US); INTAS ECL CHEMOSTAR (INTAS, Göttingen, Germany); BZ-X 810 microscope (Keyence, Osaka, Japan); TCS SP8 confocal system (Leica Microsystems, Wetzlar, Germany); Polarographic oxygen sensor (O2K-fluo, Oroboros Instrument, Innsbruck, Austria); Digital inverted microscope (iMIC, FEI Germany); UHPLC system (Vanquish Flex, Thermo Fisher Scientific, Germany) equipped with an Ascentis® Express C18 column (150 x 2.1 mm: 2.7 µm, 90 Å, Supelco, Germany) coupled to a HESI-orbital trapping mass spectrometer (Exploris 240, Thermo Fisher Scientific, Germany); StepOnePlus (Applied Biosystems, Foster City, CA, USA)
Data analysis	Vevo Lab analysis software (Version 3.2.0; FUJIFILM Visualsonics, Inc., Toronto, ON, Canada); Circlab (v12.5, Prof. P. Steendijk, Leiden University); Proteome Discoverer 2.5 software (Thermo Fisher, Dreieich, Germany); Perseus software tool version 2.0.9.0 (MPI Biochemistry); VessEval (https://github.com/pLeminoq/vesseval, Tamino Huxohl); LabImage Version 4.2.1 (Kapelan Bio-Imaging GmbH), Fiji (Version 2.14.0/1.54f); GNU Image Manipulation Program (GIMP, Version 2.10.34); Prism 10 for Windows (GraphPad, San Diego, CA, USA); Lipostar2 (Molecular Discovery, Italy)

For manuscripts utilizing custom algorithms or software that are central to the research but not yet described in published literature, software must be made available to editors and reviewers. We strongly encourage code deposition in a community repository (e.g. GitHub). See the Nature Portfolio guidelines for submitting code & software for further information.

Data

Policy information about availability of data

All manuscripts must include a data availability statement. This statement should provide the following information, where applicable:

- Accession codes, unique identifiers, or web links for publicly available datasets
- A description of any restrictions on data availability
- For clinical datasets or third party data, please ensure that the statement adheres to our policy

All data generated or analysed during this study are included in this published article and its supplementary information files. Source data are provided in a source data file. The mass spectrometry proteomics data have been deposited to the ProteomeXchange Consortium via the PRIDE partner repository with the dataset identifier PXD058165; ProteomeXchange title: Nitro-oleic acid in a HFpEF two-hit mouse model of high-fat diet and L-NAME [https://proteomecentral.proteomexchange.org/ui?search=PXD058165].

Research involving human participants, their data, or biological material

Policy information about studies with human participants or human data. See also policy information about sex, gender (identity/presentation), and sexual orientation and race, ethnicity and racism.

Reporting on sex and gender	n/a
Reporting on race, ethnicity, or other socially relevant groupings	n/a
Population characteristics	n/a
Recruitment	n/a
Ethics oversight	n/a

Note that full information on the approval of the study protocol must also be provided in the manuscript.

Field-specific reporting

Please select the one below that is the best fit for your research. If you are not sure, read the appropriate sections before making your selection.

☒ Life sciences ☐ Behavioural & social sciences ☐ Ecological, evolutionary & environmental sciences

For a reference copy of the document with all sections, see nature.com/documents/nr-reporting-summary-flat.pdf

Life sciences study design

All studies must disclose on these points even when the disclosure is negative.

Sample size	For all animal studies the sample size for the experimental group was determined based on expected differences in target parameters and their variation. The deviating sample size in-between experimental groups is explained by loss of animals either due to technical difficulties or disease burden.
Data exclusions	No data were excluded, except for Fig. 2g. One outlier was identified using ROUT method as indicated in the figure legend.
Replication	Phenotypic data were reproduced with two additional independent cohorts of mice. Main findings from proteome analyses were reproduced by immunoblot. Reproducibility of transmission electron microscopy was verified through analyzing samples from two independent cohorts. Main immunoblot analyses (Fig. 4c, 5c and suppl. figures) were successfully done with at least one technical replicate. All molecular biological and proteinbiochemical analyses were run with at least 6 biological replicates (animals) per group. Functional analyses (mitochondrial respiration of cardiomyocytes and mouse exercise testing) cannot be done with technical replicates because the tests with living animals or living cells can only be perfomed once. However, at least 5 biological replicates (animals) per group were used.
Randomization	Allocation of mice into experimental groups was random, except for allocation of mice after 11 weeks of HFD+L-NAME into treatment groups. Here, mice were allocated so that mean body weight was equal in all groups.
Blinding	All investigators were blinded to group allocation, except for the identification of chow or HFD+L-NAME while performing echocardiography, hemodynamic measurements and exercise test. Here, differences in body weight were obvious. However, the treatment of HFD+L-NAME with vehicel, OA or NO2OA was concealed during these experiments as well.

Reporting for specific materials, systems and methods

We require information from authors about some types of materials, experimental systems and methods used in many studies. Here, indicate whether each material, system or method listed is relevant to your study. If you are not sure if a list item applies to your research, read the appropriate section before selecting a response.

Materials & experimental systems

n/a	Involved in the study
☐	☒ Antibodies
☒	☐ Eukaryotic cell lines
☒	☐ Palaeontology and archaeology
☐	☒ Animals and other organisms
☒	☐ Clinical data
☒	☐ Dual use research of concern
☒	☐ Plants

Methods

n/a	Involved in the study
☒	☐ ChIP-seq
☒	☐ Flow cytometry
☒	☐ MRI-based neuroimaging

Antibodies

Antibodies used

cytochrome-c-oxidase subunit 4 (1:5000, #4844, Cell Signaling, Danvers, MA, USA), sirtuin 3 (1:1000, #5490, Cell Signaling, Danvers, MA, USA), sirtuin 1 (1:5000; #9475, Cell Signaling, Danvers, MA, USA) phosphorylation at threonine 172 of AMP-activated protein kinase (1:500, #2531, Cell Signaling, Danvers, MA, USA), AMP-activated protein kinase (1:1000, #2532, Cell Signaling, Danvers, MA, USA), phosphorylation at serine 293 of pyruvate dehydrogenase (1:1000, #31866, Cell Signaling, Danvers, MA, USA), translocase of outer mitochondrial membrane 70 (1:2000, #14528-1-AP, Proteintech, Rosemont, Il, USA), pyruvate dehydrogenase kinase 4 (1:1000, ab214938, abcam, Cambridge, UK), peroxiredoxin-SO2/3 (1:1000, CRB2005004, Biosynth, Staad, Switzerland), phosphorylation of serine 239 of vasodilator-stimulated phosphoprotein (1:1000, #3114, Cell Signaling, Danvers, MA, USA) and vasodilator-stimulated phosphoprotein (1:2000, #3112, Cell Signaling, Danvers, MA, USA), smooth muscle actin (A2547, 1:900, Sigma-Aldrich), von-Willebrand factor (PA-16634, 1:100, Invitrogen)

Validation

The peroxiredoxin-SO2/3 antibody was validated in primary adult murine cardiomyocytes exposed to different hydrogenperoxid concentrations. To validate the AMP-activated protein kinase antibody detecting the phosphorylation site of AMPK, protein samples were dephosphorylated with phosphoatase in vitro. The von-Willebrand factor antibody was validated by Wang et al. 2021 (https://doi.org/10.1016/j.ebiom.2021.103632). For all other primary antibodies validation in manufacturer's instructions were available. In detail, COX4 and p-VASP antibodies were validated in NIH/3T3 cells; Sirt3 antibody was validated in murine liver lysates; TOM70 and PDK4 antibodies were validated in murine heart tissue; SIRT1 was validated in murine embryonal fibroblasts; AMPK and VASP antibodies were validated in C2C12 cells; p-PDH antibody was validated in murine BaF3 cells.

Animals and other research organisms

Policy information about studies involving animals; ARRIVE guidelines recommended for reporting animal research, and Sex and Gender in Research

Laboratory animals

3-week-old male C57BL/6N mice were purchased from Charles River (Sulzfeld, Germany) and Janvier Labs (Le Genest-Saint-Isle, France). After 1 week of acclimatization, the animal protocols were started in 4-week old male mice. 8-week old female C57BL/6N mice were provided by in-house breeding.

Wild animals

The study did not involve wild animals.

Reporting on sex

The main findings of the manuscript reporting positive effects of nitro-oleic acid treatment on cardiac function could be confirmed in female mice, affirming the therapeutic efficiency of our component in both sexes.

Field-collected samples

The study did not involve samples collected in the field.

Ethics oversight

All animal studies were approved by the local Animal Care and Use Committees (Ministry for Environment, Agriculture, Conservation and Consumer Protection of the State of North Rhine-Westphalia: State Agency for Nature, Environment and Consumer Protection (LANUV), NRW, Germany) and followed guidelines from Directive 2010/63/EU of the European Parliament on the protection of animals used for scientific purposes.

Note that full information on the approval of the study protocol must also be provided in the manuscript.

Plants

Seed stocks	n/a
Novel plant genotypes	n/a
Authentication	n/a
